# Engineering a high-sugar tolerant strain of *Saccharomyces cerevisiae* for efficient trehalose production using a cell surface display approach

**DOI:** 10.1186/s40643-024-00816-x

**Published:** 2024-10-18

**Authors:** Kan Tulsook, Piyada Bussadee, Jantima Arnthong, Wuttichai Mhuantong, Panida U-thai, Srisakul Trakarnpaiboon, Verawat Champreda, Surisa Suwannarangsee

**Affiliations:** National Center for Genetic Engineering and Biotechnology (BIOTEC), National Science and Technology Development Agency (NSTDA), 113 Thailand Science Park, Klong Luang, Pathumthani, 12120 Thailand

**Keywords:** Trehalose production, Trehalose synthase, *Saccharomyces cerevisiae*, Cell surface display, Maltose, *Acidiplasma aeolicum*

## Abstract

Trehalose production via a one-step enzymatic route using trehalose synthase (TreS) holds significant promise for industrial-scale applications due to its simplicity and utilization of low-cost substrates. However, the development of a robust whole-cell biocatalyst expressing TreS remains crucial for enabling practical and economically viable production. In this study, a high-sugar tolerant strain of *S. cerevisiae* was screened and employed as a host cell for the cell surface display of TreS from *Acidiplasma aeolicum*. The resultant strain, *S. cerevisiae* I3A, exhibited remarkable surface displayed TreS activity of 3358 U/g CDW and achieved approximately 64% trehalose yield (10.8 g/L/h productivity) from maltose. Interestingly, no glucose by-product was observed during trehalose production. The *S. cerevisiae* I3A cells exhibited reusability for up to 12 cycles leading to potential cost reduction of trehalose products. Therefore, our study demonstrated the development of a high-sugar tolerant *S. cerevisiae* strain expressing TreS on its surface as a whole-cell biocatalyst for efficient and economical trehalose production with potential applications in the food and pharmaceutical industries.

**Supplementary Information:**

The online version contains supplementary material available at 10.1186/s40643-024-00816-x.

## Introduction

Trehalose is a naturally occurring sugar found in more than 80 diverse living species ranging from bacteria and fungi to plants and invertebrates, where it functions as an osmoprotectant, energy source, and abiotic stress protectant (Raza et al. 2023). It is a non-reducing disaccharide consisting of two glucose units linked by an α, α-1,1-glycosidic bond. Trehalose possesses highly stable and chemically unreactive properties that resist the Maillard reaction and remain intact unless subjected to extreme hydrolytic conditions or the activity of trehalase (Schiraldi et al. 2002). Leveraging its unique characteristics, trehalose has been extensively applied as a food additive contributing to moisture retention, prolonged product shelf life, and enhanced flavor in the food industry. Additionally, it serves as a moisture-retaining agent and storage stability enhancer in the cosmetic and pharmaceutical industries (Cai et al. 2018). Recently, there has been growing interest in trehalose as a potential biomolecule for promoting human health. It has been suggested to be used as a low glycemic index sweetener for diabetes patients (Yaribeygi et al. 2019). It was also explored as a potential therapeutic agent for mitigating neurodegenerative and cardiovascular diseases (Khalifeh et al. 2021). Taken together, these findings led to an increased global demand for trehalose (Zahedi et al. 2023).

Trehalose can be synthesized by trehalose synthase (TreS, EC 5.4.99.16) which catalyzes the reversible interconversion of maltose to trehalose without requiring a coenzyme (Han et al. 2006). This single-enzyme method has garnered considerable attention due to its simplicity, high conversion yield, and suitability for industrial-scale production (Liu et al. 2019a; Zhu et al. 2020). Moreover, the maltose substrate can be obtained through the enzymatic hydrolysis of starch, a polysaccharide found abundantly in various plant sources including corn, cassava, and rice. The TreS enzymes have been reported in many bacterial species such as *Picrophilus torridus*, *Deinococcus radiodurans*, *Pseudomonas stutzeri*, *Pseudarthrobacter* sp., etc. (Chen et al. 2006; Filipkowski et al. 2012; Lee et al. 2005; Trakarnpaiboon et al. 2023). However, the expression level of trehalose synthase in naturally occurring bacterial strains is notably limited (Li et al. 2016). Numerous investigations have endeavored to improve the expression of TreS through genetic engineering. These efforts involved the heterologous expression of TreS as a free enzyme for converting maltose to trehalose (Yang et al. 2015). However, the utilization of the free form of TreS in biocatalysis was limited to single-use applications unless specialized immobilization techniques were employed (Panek et al. 2013). Recently, surface-displayed TreS in host cells like *Bacillus subtilis*, *Corynebacterium glutamicum*, and *Komagatella phaffii* (*Pichia pastoris*) have emerged as promising whole-cell biocatalysts. This approach offers the potential for reusability, thereby potentially leading to significant cost reductions in trehalose production (Fang et al. 2023; Liu et al. 2019b; Yang et al. 2017).

Within the array of expression systems, yeast cell surface display emerges as a promising and straightforward method for efficient trehalose production. The yeast cell surface display (YSD) technique involves immobilizing the target enzyme onto the yeast cell surface through fusion with an anchoring protein motif (Phienluphon et al. 2019). This method offers several advantages including a simple process, enhanced enzyme stability, self-immobilization of enzymes during cell cultivation, and the recyclability of the surface-anchored enzyme (Arnthong et al. 2022; Lozančić et al. 2019). Previously, TreS from *Pseudomonas putida* ATCC47054 displaying on the *P. pastoris* cell surface was able to produce trehalose from maltose syrup up to 64% of conversion yield with recycling performed three times (Yang et al. 2017). Another investigation reported the development of a *Y. lipolytica* strain expressing *P. torridus* TreS on the cell surface with 73% of trehalose production yield and 4.5 g/L/h of productivity, however, the recycling capacity was unexplored (Li et al. 2016).

Despite the success achieved in these host systems, there are still challenges including biocatalyst stability and recycling efficiency which endure significance in the context of the overall production cost of trehalose. The consideration of food safety issues is also necessary for industrial trehalose production (Cai et al. 2018). *S. cerevisiae* is a well-established industrial microorganism employed in food and alcoholic fermentation applications (Liu et al. 2022). It is classified as GRAS (“generally recognized as safe”) and does not generate endotoxins like those produced by *E. coli* that may exert adverse effects on human health (Wallace et al. 2016). Furthermore, the expression of heterologous proteins in *S. cerevisiae* eliminates the need for adding isopropyl β- d-1-thiogalactopyranoside (IPTG), methanol, or other toxic agents during fermentation. This advantage facilitates the application of the resulting products in the food, pharmaceutical, and agricultural industries (Zhang and Cao 2024).

During trehalose production, a high maltose concentration of 300 g/L is commonly utilized, which can induce stress and compromise the stability of the yeast whole-cell biocatalyst. In this study, we screened *S. cerevisiae* strains for their ability to tolerate high concentrations of maltose. The yeast strain demonstrating the maximum tolerance was selected as the host strain for the development of a whole-cell biocatalyst for trehalose production. Subsequently, the compatibility of different TreS enzymes for tethering to the yeast cell wall was investigated. To optimize trehalose production, different bioprocess parameters including pH, temperature, concentrations of yeast cells, and concentration of maltose substrate were studied. The reusability of TreS-displayed yeast cells was also evaluated. Finally, the scalability of trehalose production was assessed using a 5-L bioreactor. To the best of our knowledge, this is the first report on the utilization of a high-sugar tolerant *S. cerevisiae* strain expressing TreS on its surface for trehalose production with high potential for industrial applications.

## Materials and methods

### Strains and culture conditions

*S. cerevisiae* strains were obtained from the Thailand Bioresource Research Center (TBRC, www.tbrcnetwork.org) for examination of high-sugar tolerant characteristics. *S. cerevisiae* TBRC 3611 was used as a host strain for the cell surface display of TreS. Yeast strains were maintained in yeast peptone dextrose (YPD) agar medium (1% w/v yeast extract, 2% w/v peptone, 2% w/v glucose, and 1.5% w/v agar) at 30 °C. Yeast transformants were grown in YPD medium supplemented with 100 µg/mL G418 or synthetic complete (SC) medium (0.67% w/v yeast nitrogen base without amino acids, 2% w/v glucose, and 1.5% w/v agar) with the required amino acid drop-out supplement (Takara Bio, Shiga, Japan). For the expression of TreS, recombinant yeast cells were cultivated in YPD medium at 30 °C with shaking at 200 rpm for 48 h. *E. coli* DH5α (Invitrogen, Carlsbad, CA, USA) was used for DNA manipulation and plasmid propagation. The bacteria were maintained in Luria–Bertani medium (1% w/v tryptone, 0.5% w/v yeast extract, and 1% w/v NaCl). The *E. coli* transformant was selected on LB medium with 100 µg/mL ampicillin.

### Determination of yeast cell viability

Yeast cells were cultured in 10 mL of YPD medium at 30 °C with shaking at 200 rpm for 16–18 h. Then, the cells were washed twice with sterile distilled water and resuspended with 50 mM sodium phosphate buffer pH 6.0 containing 300 g/L maltose to obtain the final cell concentration of 5 OD_600_/mL. The cell suspension was incubated at 30 °C with shaking at 200 rpm for 72 h. Yeast cell samples were collected every 24 h for determination of cell viability by using methylene violet staining method following the previous report (Smart et al. 1999).

### Sugar consumption test

To evaluate the sugar consumption capabilities of *S. cerevisiae* TBRC 3611, the overnight grown cells were washed twice with sterile distilled water and resuspended in 10 mL of YPD, YPT (2% w/v trehalose), or YPM (2% w/v maltose) medium to achieve an initial cell concentration of 0.01 OD_600_/mL. The cell cultures were cultivated at 30 °C with shaking at 200 rpm. Samples were collected at 0, 6, 12, 18, and 24 h and were centrifuged at 7,000 *g*. The concentrations of glucose, maltose, or trehalose remaining in the culture media were analyzed by using HPLC.

### Plasmid construction and yeast transformation

For screening of *treS* genes, the episomal vector pYES3-Kan, a variant of the pYES3/CT vector (Invitrogen, USA) containing the G418 resistance gene (KanMX) as a selectable marker was obtained from the laboratory’s stock and employed as a vector for gene expression. The *GPD1* promoter (GPD1p), a signal peptide from *SUC2* gene (SS_suc2_), codon-optimized *Kluyveromyces lactis PIR4* (KlPIR4) mature sequences, and the codon-optimized *treS* gene from *P. torridus* DSM9790 (PtTreS) were chemically synthesized and cloned into pYES3-Kan at the *Spe*I-*Xho*I site resulting the yeast surface display vector pYk-KlPIR4-PtTreS. The plasmid pYk-KlPIR4-PtTreS was then used as the backbone for cloning of different codon-optimized *treS* genes (Table S1) in place of PtTreS at the *Eco*RI/*Bam*HI site, yielding the desired surface display expression vector. The recombinant plasmid was transformed into *S. cerevisiae* TBRC 3611 following an established protocol (Helmuth et al. 2001). The positive clones were determined by using colony PCR and DNA sequencing methods.

For stable and high-level expression of TreS, a multi-copy integrative vector pYIR3 was used as a backbone vector. The vector pYIR3 consisted of the *ADE2* selection marker gene and can be integrated into the ribosomal DNA (rDNA) site of the yeast genome. The gene expression cassette containing GPD1p, SS_suc2_, KlPIR4, and codon-optimized *treS* gene from *Acidiplasma aeolicum* (AaTreS) were linearized from pYk-KlPIR4-AaTreS using *Spe*I/*Xho*I digestion and then ligated into pYIR3 at *Xba*I/*Xho*I site, yielding the pYIR3-AaTreS. The pYIR3-AaTreS plasmid was linearized with *Pme*I and was transformed into *S. cerevisiae* TBRC3611-ΔADE2. The positive clones were determined by colony PCR. Strains, plasmids, and primers used in this study are listed in Table S2 and S3, respectively.

### Assay for the surface-display TreS activity

The yeast cells expressing TreS were collected by centrifugation at 8,000 *g* and the supernatant was discarded. The cells were washed twice with 50 mM sodium phosphate buffer pH 6.0. The enzymatic reactions (1 mL) were composed of 1 OD_600_ yeast cells, 300 g/L maltose in 50 mM sodium phosphate buffer pH 8.0. The reaction tubes were incubated at 30 °C with shaking at 200 rpm for 9 h. After that, cells were removed by centrifugation at 8,000 *g* for 10 min. The amount of trehalose was estimated by using the trehalose assay kit (Megazyme, Bray, Ireland) (Trakarnpaiboon et al. 2021). One unit of trehalose synthase refers to the amount of enzyme that produces 1 µmol of trehalose from maltose per hour under the assay conditions. One unit of OD_600_ was equivalent to 0.459 g of cell dry weight (CDW)/L. The experiments were performed in triplicate.

### Immunofluorescent assay

Yeast cells were harvested and washed twice with phosphate-buffered saline (PBS, pH 7.0) before undergoing immunofluorescence labeling following a previously published protocol (Phienluphon et al. 2019). A mouse monoclonal anti-His antibody was used as the primary antibody, and an Alexa Fluor 647-conjugated anti-mouse IgG antibody served as the secondary antibody. Fluorescent signals were observed using a confocal laser scanning microscope (FluoView FV1000, Japan) with excitation and emission wavelengths of 635 nm and 668 nm, respectively. Images were acquired and processed with Fluoview 3.0 software (Olympus).

### Characterization of the surface displayed TreS

The yeast cells expressing TreS were collected by centrifugation at 8,000 *g* and the supernatant was discarded. The cells were washed twice with 50 mM sodium phosphate buffer pH 8.0 and used for enzyme characterization. To determine the effect of temperature on the displayed TreS activity, the assay was examined under the same conditions as described above at various temperatures (20–50 °C). For the effect of pH, the TreS activity assay was conducted at 30 °C by using 50 mM sodium acetate buffer (pH 4.0–5.0), sodium phosphate buffer (pH 6.0–8.0), or sodium carbonate buffer (pH 9.0–10.0).

To determine the thermostability of the surface-displayed TreS, the yeast cells were resuspended in 50 mM sodium phosphate buffer pH 8.0 and were incubated at various temperature ranges from 30 to 80 °C for 1 h. After that, the cells were collected by centrifugation and were examined for the TreS activity under the standard condition. To evaluate the pH stability of the displayed TreS, the yeast cells were resuspended in a buffer with pH ranging from 4.0 to 10.0 for 1 h. The cells were then collected for the determination of TreS activity under the standard condition.

To study the influence of glucose on the surface-displayed TreS activity, the activity assay was conducted under standard conditions except that the assay solution was supplemented with varying concentrations of glucose ranging from 0 to 40 g/L.

To assess the storage stability of the surface-displayed TreS activity, yeast cells were resuspended in a 50 mM sodium phosphate buffer solution at pH 8.0 to yield an OD_600_ of 10. The cell suspension was stored at 4 °C. The cells were withdrawn from the storage weekly for 4 weeks to determine the TreS activity under standard conditions.

### Optimization of trehalose production by yeast whole-cell biocatalyst

The yeast cells were cultivated in 50 mL of the fermentation medium containing 37 g/L sucrose, 15 g/L yeast extract, and basal medium following previously reported (Wang et al. 2007). The cell cultivation was performed at 33 °C for 48 h. The cells were collected and washed twice with 50 mM sodium phosphate buffer pH 8.0. To assay for optimal cell concentration, the yeast cells were resuspended in 300 g/L maltose solution to obtain the final concentration of 10–50 OD_600_/mL. The reaction was performed at 40 °C with shaking at 200 rpm for 24 h. To assay for optimal substrate concentration, the yeast cell concentration of 20 OD_600_/mL was resuspended in 50–500 g/L maltose solution. The assay conditions were carried out at 40 °C at 200 rpm for 24 h. The supernatant was collected and the sugar concentration was analyzed using HPLC.

### Reusability of TreS-displayed yeast cells

To assess the reusability of TreS-displayed yeast cells, their ability to undergo multiple trehalose production cycles was investigated. Initially, the yeast cells were resuspended in 25 mL of a 300 g/L maltose solution to reach a final concentration of 20 OD_600_/mL. This suspension was then incubated at 40 °C with shaking at 200 rpm for 24 h. After incubation, the cells were separated from the supernatant by centrifugation. The supernatant was stored at -20 °C for sugar analysis by HPLC. The collected yeast cells from the first cycle were collected and resuspended in fresh 25 mL of 300 g/L maltose solution, initiating the next trehalose production cycle. All subsequent cycles (up to 14) were carried out under identical conditions to the first cycle. The experiment was performed in triplicate.

### Yeast cell production and trehalose production in a 5-L bioreactor

For the preparation of TreS displaying yeast cells, *S. cerevisiae* strain I3A was cultivated in a 5-L bioreactor (Biostat B, Sartorius, Germany). The fermentation medium containing 37 g/L sucrose, 15 g/L yeast extract, and basal medium following previously reported (Wang et al. 2007) was prepared and adjusted to pH 7.0 with a media volume of 4 L, with the addition of 0.01% v/v of antifoam. After sterilization, the fermentation medium was inoculated with 5% (v/v) yeast inoculum. The temperature was maintained at 33 °C, an agitation rate of 250 rpm, an aeration rate of 1.0 vvm, without pH control. Samples were taken every 12 h until 48 h. The yeast cells were harvested and centrifuged at 5,000 *g* for 10 min at 4 °C and further studied for trehalose production in bioreactors.

Trehalose production was performed in a 5-L bioreactor with a working volume of 2.5 L containing 300 g/L maltose in 50 mM sodium phosphate buffer pH 8.0 and sterilized at 121 °C for 15 min. After that, the yeast cells were added to obtain the final concentration of 20 OD_600_/mL. The fermentation was performed at 40 °C, an agitation rate of 200 rpm, and an aeration rate of 0.5 vvm for 48 h. Samples were taken every 3–12 h until 42 h for sugar analysis by HPLC. The maltose-to-trehalose conversion rate was determined using maltose and trehalose concentrations that were quantified by HPLC analysis.

### Sugar analysis

The concentration of glucose, maltose, and trehalose in the samples was analyzed using an HPLC system equipped with Shodex VG-50 4E column (Resonac Corp., Tokyo, Japan) and refractive index detector (RID) at a flow rate of 1.0 mL/min at 40 °C. The column was equilibrated with 80% acetonitrile and 20% pure water. The sugar concentration in the sample was compared to the standard curves of its respective substances.

## Results and discussion

### Screening of a high-sugar tolerant *S. cerevisiae* strain

**Fig. 1:**
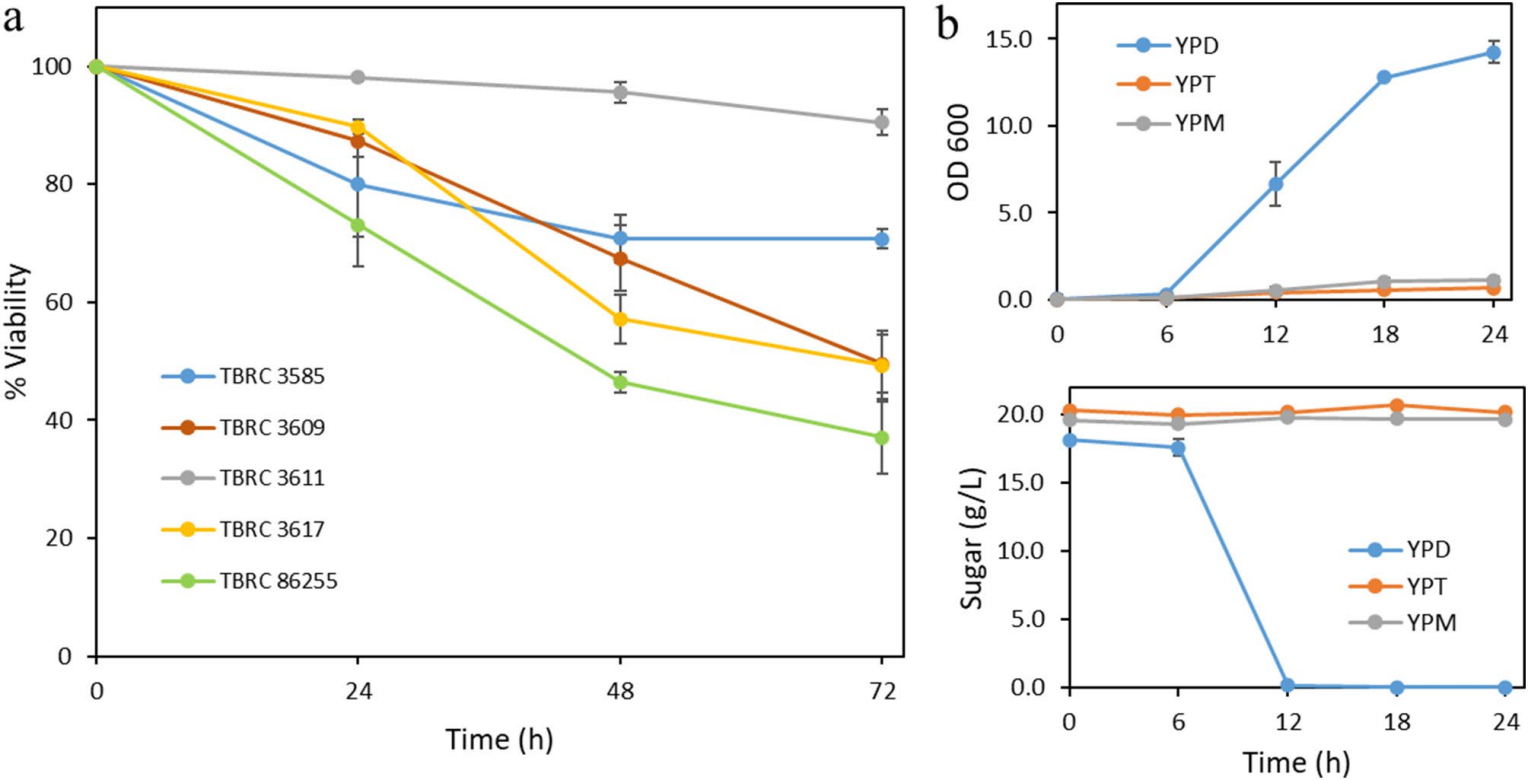
(**a**) Viability of yeast cells in 300 g/L of maltose solution after incubation for 24, 48, and 72 h. (**b**) Cell growth and residual sugar concentration after cultivation of *S. cerevisiae* TBRC 3611 in YPD, YPM, or YPT. Data represent an average of three independent experiments. Error bars indicate standard deviation (SD)

To obtain a *S. cerevisiae* host strain with high sugar tolerance properties, a preliminary screening was conducted on various wild-type *S. cerevisiae* strains for their growth ability on agar plates containing 30% (w/v) glucose (data not shown). From this screening, five strains namely TBRC 3585, TBRC 3609, TBRC 3611, TBRC 3617, and TBRC 86,255 were chosen. The capacity of these *S. cerevisiae* strains to withstand high maltose concentrations was examined by incubation of the cells in 300 g/L maltose solution followed by an assessment of cell viability. Among these strains, TBRC 3611 exhibited notable cell viability of 91% after a 72-h incubation in the 300 g/L maltose solution (Fig. 1a). Subsequently, the sugar consumption patterns of the TBRC 3611 strain were investigated over a 24-h period in three different liquid media: YPD, YPT, and YPM (Fig. 1b). It was found that the TBRC 3611 displayed limited growth in YPT and YPM media when compared to YPD medium. Furthermore, there was no significant alteration in maltose and trehalose concentrations in the YPM and YPT media after 24-h incubation. This finding suggests limited utilization of maltose and trehalose by the TBRC 3611 strain during this timeframe, making it suitable for application in the trehalose production from maltose substrate. Based on these observations, the *S. cerevisiae* TBRC 3611 strain was selected as a host for constructing a yeast whole-cell biocatalyst. The auxotrophic Ade2∆ mutant of this strain was created by using a homologous recombination approach.

### Screening of trehalose synthase for yeast cell surface display

**Fig. 2:**
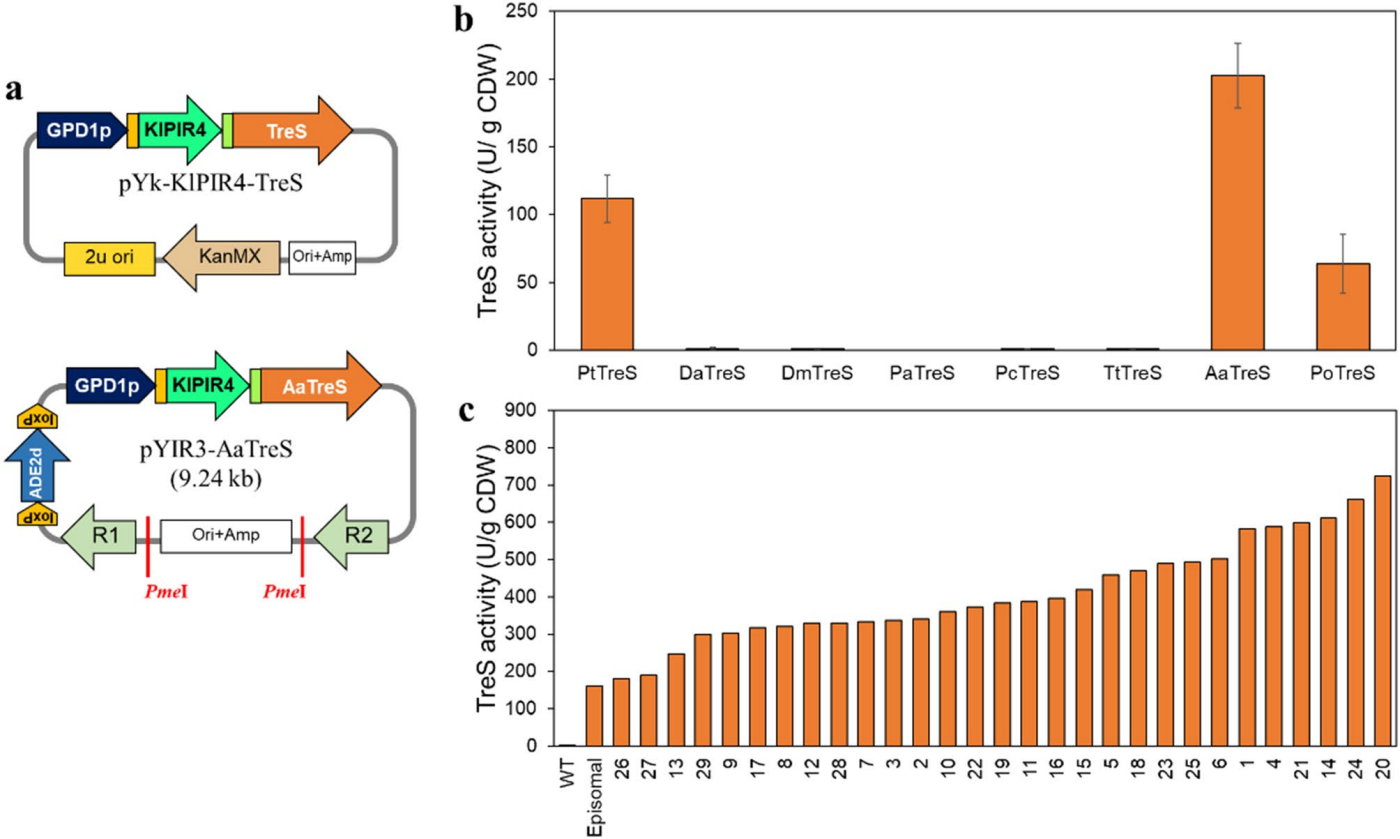
(**a**) Map of the episomal plasmid pYk-KlPIR4-TreS and the multi-copy integration plasmid pYIR3-AaTreS. (**b**) Surface-displayed TreS activity of the yeast cells carrying the pYk-KlPIR4-TreS containing different *treS* genes. Data represent an average of five independent experiments. Error bars indicate SD. (**c**) Surface-displayed TreS activity of the yeast cells carrying pYIR3-AaTreS. The activity of yeast cells carrying pYk-KlPIR4-AaTreS (Episomal) and TBRC 3611 (WT) were applied as a control. The experiments were performed using one biological sample for each clone

Based on our previous report, the surface displayed cellulolytic enzyme activities on *S. cerevisiae* varied depending on the enzyme sources (Arnthong et al. 2022). To identify TreS enzymes suitable for display on the yeast cell wall, *treS* genes from diverse microbial origins were explored. These *treS* genes were fused with *KlPIR4*, a *K. lactis* ortholog gene to *S. cerevisiae* PIR4 at the N-terminus to facilitate the immobilization of TreS enzymes onto the yeast cell wall (Varela et al. 2019) (Fig. 2a). The recombinant vectors containing different *treS* genes were introduced into *S. cerevisiae* TBRC 3611 cells to generate recombinant yeast strains expressing TreS on their surface. We found that PtTreS from *P. torridus*, AaTreS from *Acidiplasma aeolicum*, and PoTreS from *Picrophilus oshimae* exhibited surface-displayed TreS activity within the range of 77 to 203 U/g CDW, while DaTreS from *Deinococcus aquatilis*, DmTreS from *Deinococcus murrayi*, PaTreS from *Pseudomonas azotifigens*, PcTreS from *Pseudomonas cichorii*, and TtTreS from *Thermus thermophilus* exhibited a lack of surface-displayed TreS activity (Fig. 2b). The absence of activity of certain heterologous TreS enzymes may be attributed to several factors such as mRNA secondary structure, protein misfolding, or post-translational regulatory mechanisms that modulate enzyme activity levels (Yang et al. 2022). Notably, among all TreS examined in this study, the AaTreS demonstrated the highest TreS activity of 203 ± 24 U/g CDW on the yeast cell surface.

To enhance both genetic stability and expression level of AaTreS on the yeast cell surface, the KlPIR4-AaTreS gene cassette underwent subcloning into a multi-copy integration vector yielding pYIR3-AaTreS plasmid. After transformation into *S. cerevisiae* TBRC 3611-Ade2Δ strain, 29 positive transformants were screened for enzymatic activity. It was found that the clone 20 exhibited the surface displayed TreS activity up to 724 U/g CDW, corresponding to 4.5 times higher than AaTreS expressed from the episomal vector (161 U/g CDW) (Fig. 2c). Subsequently, the clone 20 was designated as the I3A strain and used for all further experiments.

### Characterization of the surface displayed AaTreS in *S. cerevisiae* I3A

**Fig. 3:**
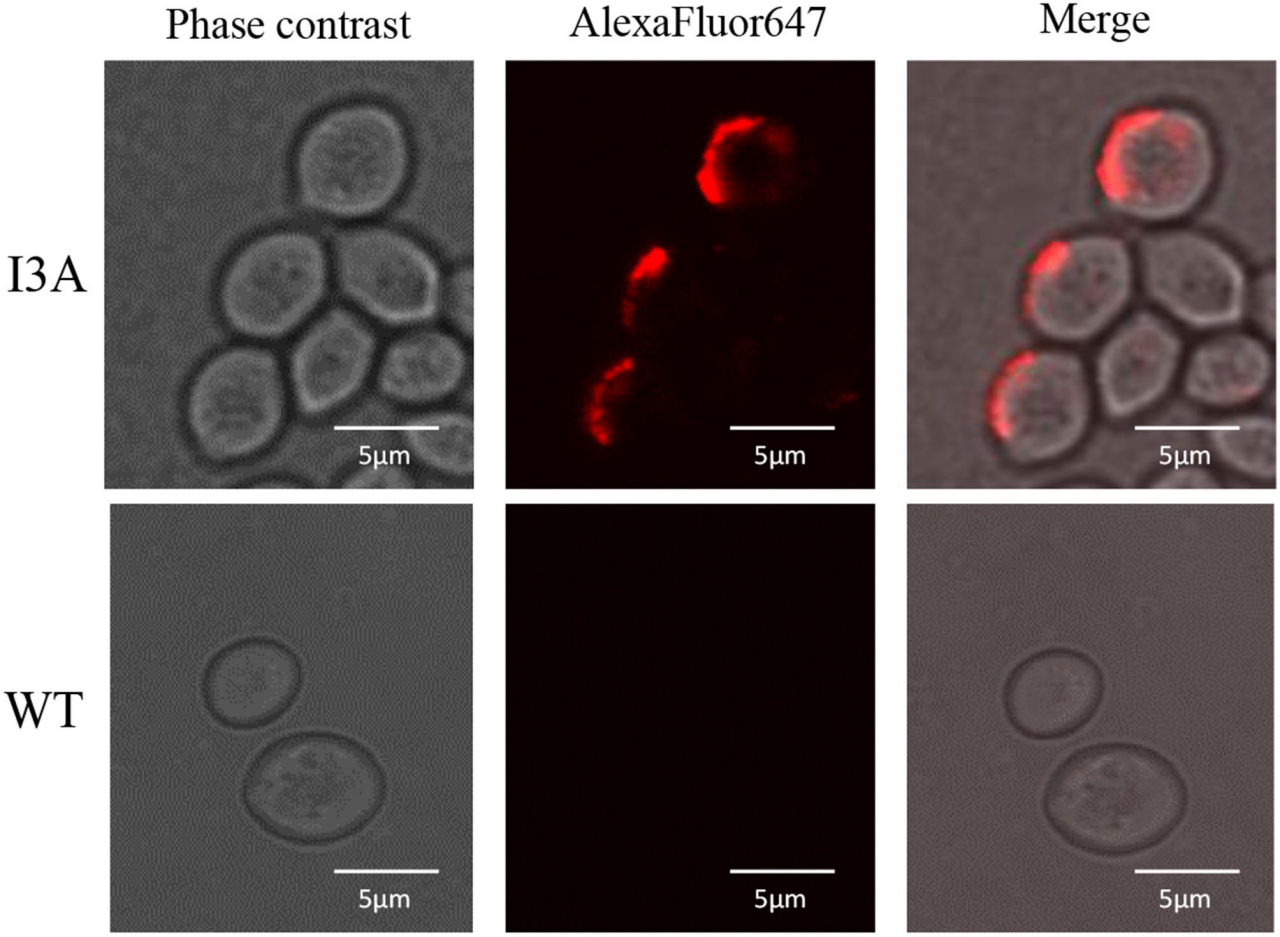
Immunofluorescent detection of AaTreS fusion proteins on the *S. cerevisiae* I3A cell surface. Mouse anti-His monoclonal antibody and goat anti-mouse IgG conjugated with Alexa Flour Plus 647 dye were applied for immunolabelling. *S. cerevisiae* TBRC 3611 (WT) cells were applied as a negative control

To confirm the expression of the AaTreS fusion protein on the yeast cell wall, immunofluorescence analysis was performed. From Fig. 3, red fluorescent signals were observed on the surface of *S. cerevisiae* I3A cells, whereas no fluorescent signal was detected on the control *S. cerevisiae* TBRC 3611 cells. These results indicate the successful expression and localization of the AaTreS enzyme on the yeast cell surface.

**Fig. 4:**
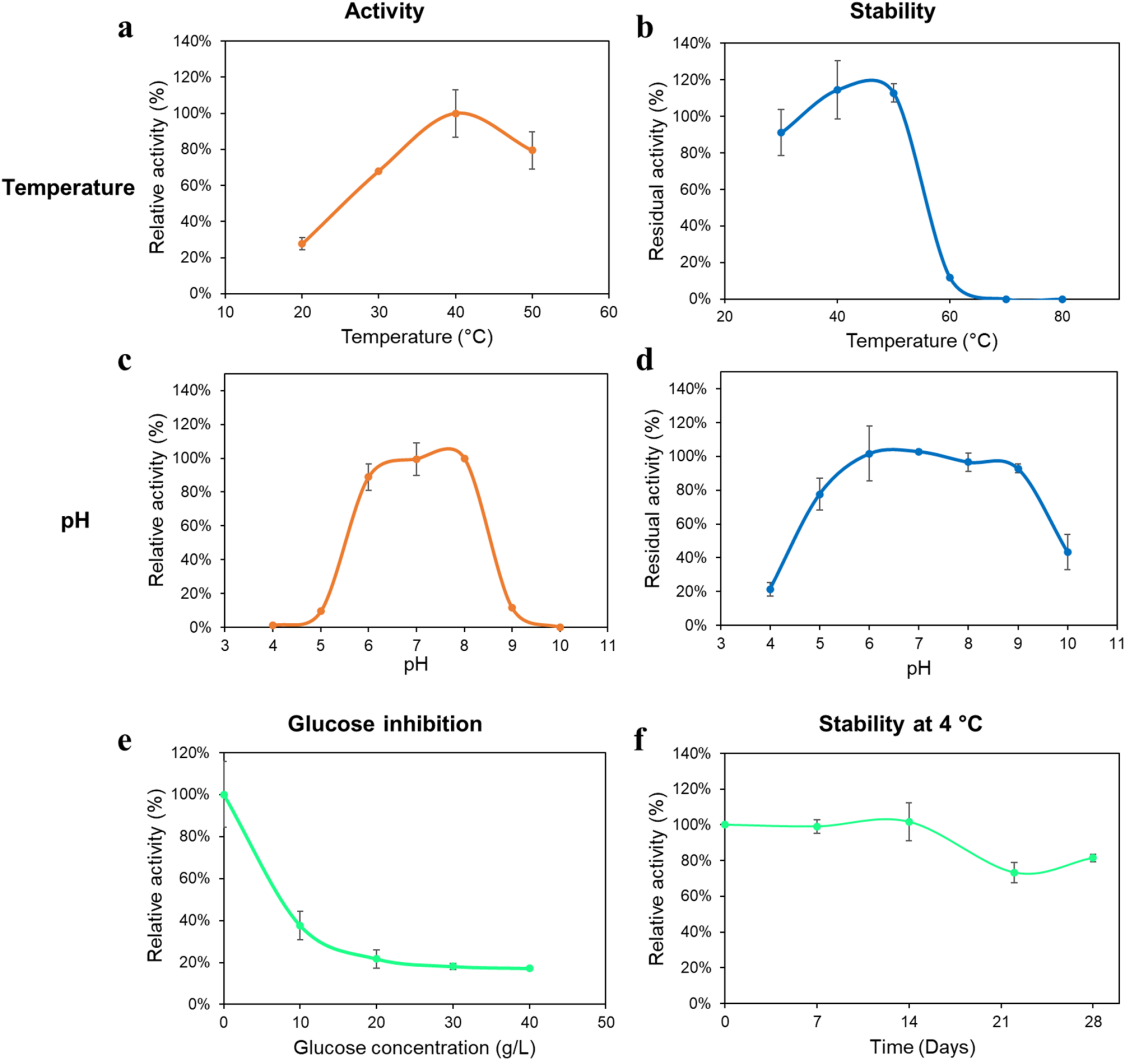
Properties of the surface-displayed AaTreS in *S. cerevisiae*. (**a**) Effect of temperature. The activity at 40 °C was defined as having a relative activity of 100%. (**b**) Thermal stability. The residual activity was compared to a sample with no heat treatment. (**c**) Effect of pH. The activity at pH 8.0 was defined as having a relative activity of 100%. (**d**) pH stability. The cells were incubated in buffers at various pH levels for 1 h at 30 °C and the activity was tested at pH 8.0. The residual activity was compared to a sample with no pH treatment. (**e**) Effect of glucose on the surface-displayed AaTreS in *S. cerevisiae*. The activity at 0 g/L glucose was defined as having a relative activity of 100%. (**f**) Effect of storage time on the surface-displayed AaTreS in *S. cerevisiae*. The relative activity was compared to the initial activity. Data represent an average of three independent experiments. Error bars indicate SD

To determine the optimum temperature and pH for the displayed AaTreS in *S. cerevisiae* whole cells, the TreS activity was investigated at different temperatures and pH conditions. Figure 4a shows that the maximum TreS activity was achieved at 40 °C while 68.1% and 79.4% of maximum activity were detected at 30 °C and 50 °C, respectively. For thermal stability, the AaTreS-displayed yeast cells were able to retain more than 90% of its initial activity after incubation at temperatures ranging from 30 to 50 °C for 1 h. In contrast, the surface displayed AaTreS lost nearly 90% activity after incubation at 60 °C for 1 h. (Fig. 4b). Additionally, the surface displayed AaTreS on *S. cerevisiae* cells demonstrates enhanced stability at 50 °C, maintaining 110% of its initial activity. Figure 4c illustrates that the activity of the surface-displayed AaTreS remained consistent between pH 6.0 and 8.0, with peak activity observed at pH 8.0. Conversely, beyond the pH range of 9.0 to 10.0, TreS activity exhibited a decline to 0–12% of its initial activity. The pH stability assays indicated no significant decline in TreS activity following the 1-h incubation across the pH range of 6.0 to 9.0 (Fig. 4d). Compared to the previous reports, the optimum temperature and pH of the surface-displayed AaTreS obtained in this study is similar to that of the TreS enzymes from *Deinococcus geothermalis*, *Thermobaculum terrenum*, and *Acidiplasma* sp. MBA-1 (Filipkowski et al. 2012; Wang et al. 2017a; Al Faik et al. 2019).

During conversion of maltose to trehalose, glucose was observed as a by-product owing to the modest hydrolase activity exhibited by most of TreS enzymes that hydrolyze trehalose and/or maltose into glucose (Cai et al. 2018). Nevertheless, the addition of glucose molecules in the catalytic reaction of TreS was found to be disadvantageous and diminished trehalose yield (Wei et al. 2004). In this study, the effect of glucose on the surface displayed AaTreS activity of *S. cerevisiae* whole cells was examined (Fig. 4e). A glucose concentration of 10 g/L strongly inhibits the surface-displayed AaTreS activity, yielding only 38% of the initial activity. Increasing the glucose concentration to 40 g/L further reduces catalytic activity to 17% of the initial level. This result indicates that the presence of glucose in the catalytic reaction impedes trehalose production yield by the surface-displayed AaTreS, which is consistent with previous observations (Wei et al. 2004; Agarwal and Singh 2021).

The impact of storage time on the surface-displayed AaTreS activity of *S. cerevisiae* cells was investigated at 4 °C over 28 days (Fig. 4f). The engineered yeast cells maintained their original TreS activity for 14 days. After 28 days of storage, approximately 20% decline in activity was observed. Additionally, the cell morphology of *S. cerevisiae* I3A cells observed under a light microscope after 7 to 21 days of storage remained similar to their morphology at the beginning of the experiment (Figure S1). These findings suggest that the engineered yeast cells exhibit stability and can be stored for at least one month facilitating consecutive trehalose production processes.

### Trehalose production by using *S. cerevisiae* cells displaying AaTreS

**Fig. 5:**
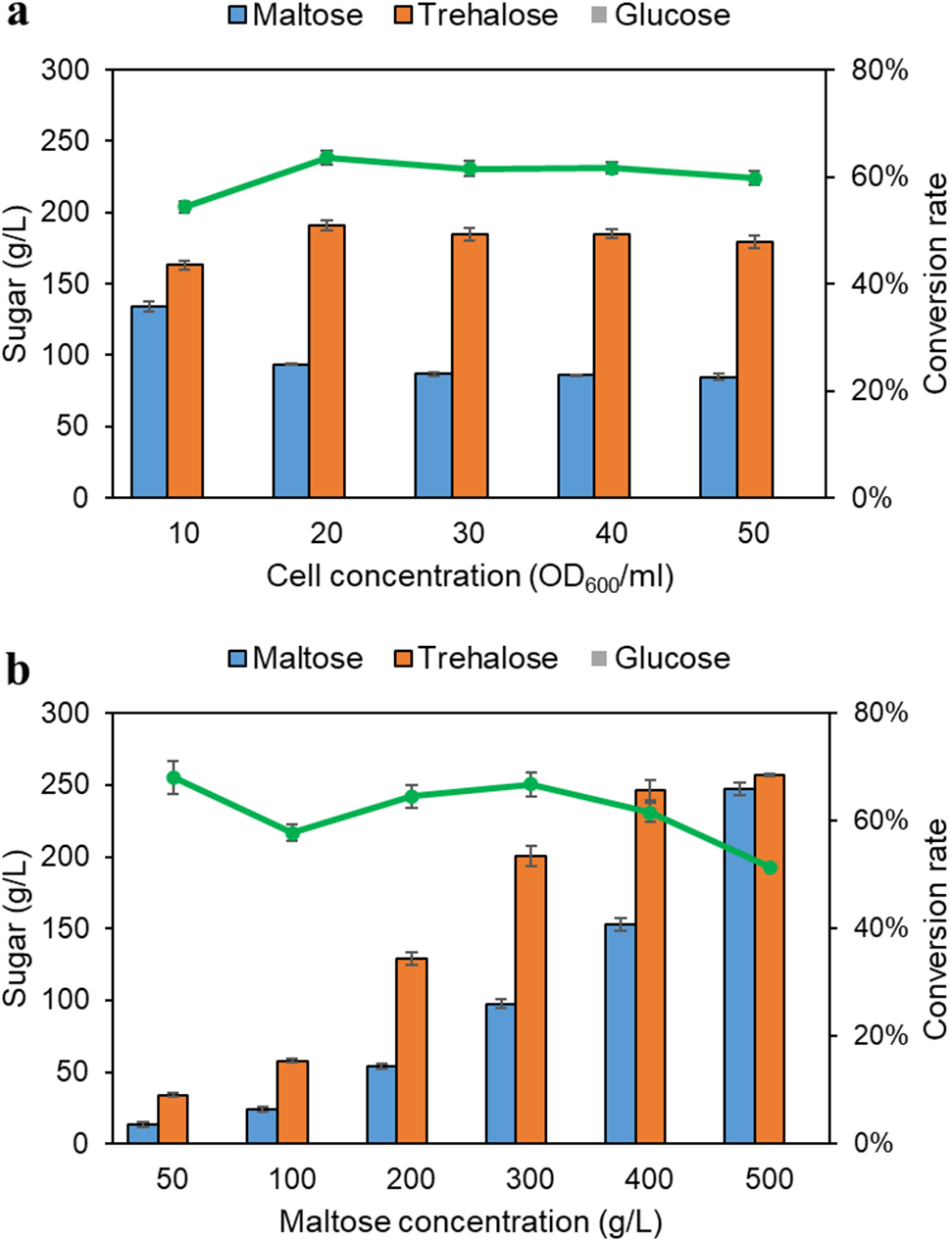
Effect of yeast cell concentration (**a**) and maltose concentration (**b**) on trehalose production by *S. cerevisiae* cells displaying AaTreS. A bar chart denotes the concentration of sugars and a line graph denotes the maltose-to-trehalose conversion rate. Data represent an average of three independent experiments. Error bars indicate SD

To enhance trehalose production, *S. cerevisiae* I3A was grown at 33 °C in 50 mL fermentation medium. After 48-h cultivation, the cells were harvested and the collected yeast cells exhibited surface-displayed TreS activity of 2485 ± 64 U/g CDW. This represents a 3.4-fold increase compared to the activity observed in YPD medium. Subsequently, the effect of various concentrations of *S. cerevisiae* I3A and maltose substrate on trehalose production was evaluated at 40 °C and pH 8.0 over a 24-h incubation period. Figure 5a represents that a conversion rate of up to 60–64% was achieved when 20–50 OD_600_/mL of the yeast cells were applied. The highest trehalose concentration of 191 ± 4 g/L was obtained from 300 g/L maltose using 20 OD_600_/mL of yeast cells. The influence of substrate concentration on trehalose production was then examined. As shown in Fig. 5b, increasing maltose concentration resulted in higher trehalose production. The maximum trehalose production of 200 ± 5 g/L, equivalent to a 67% conversion rate, was obtained with 300 g/L maltose. However, at a maltose concentration of 500 g/L, trehalose yield decreased to 51%. This reduction may be attributed to the increased viscosity caused by high maltose concentration, which could potentially interfere protein folding and enzyme (Li et al. 2016). Interestingly, glucose, a by-product catalyzed by the side hydrolysis activity of TreS was not detected in all experiments. As shown by the HPLC chromatograms of trehalose production by *S. cerevisiae* I3A cells, no glucose peak was observed in the trehalose production samples after 24-h incubation under optimized conditions (Figure S2a).

### Reusability of TreS-displayed yeast

**Fig. 6:**
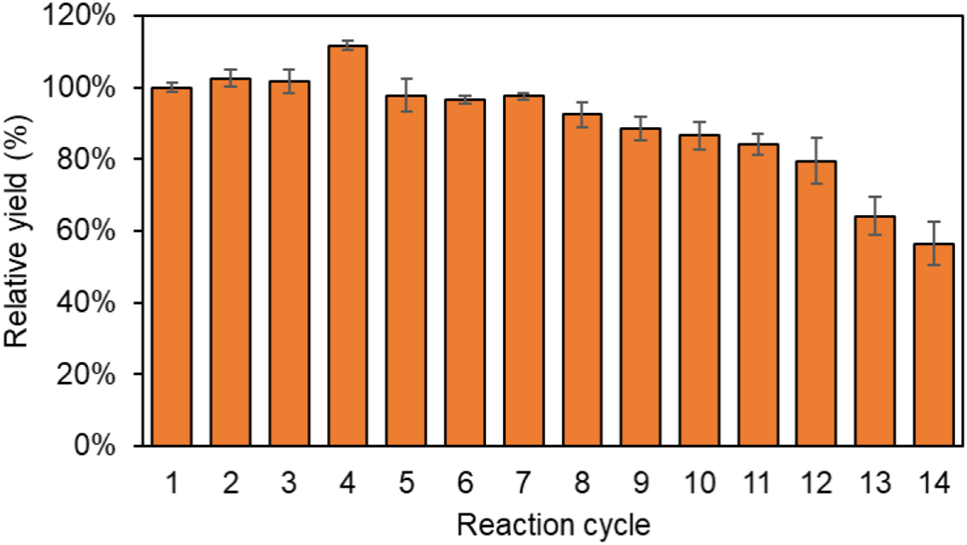
Relative trehalose production yield of *S. cerevisiae* I3A in the repeated biotransformation cycles. The trehalose yield obtained from the first cycle was defined as 100%. Data represent an average of three independent experiments. Error bars indicate SD

The reusability of *S. cerevisiae* strain I3A for trehalose production was assessed by monitoring the trehalose conversion yield from 300 g/L maltose under the previously optimized conditions. It was found that the relative trehalose production rate remained above 90% of the initial yield for the first eight cycles without increasing the conversion time (Fig. 6). In the twelfth cycle, a decline in the trehalose production rate of the yeast cells was observed, with a decrease of approximately 20%. These findings suggest that *S. cerevisiae* I3A cells can be effectively reutilized for trehalose production for up to 12 cycles with more than 80% of the initial conversion rate, potentially leading to a cost-reduction for trehalose production process. The reusability of *S. cerevisiae* I3A for trehalose production obtained from this study is better than the previous observations in other *S. cerevisiae* cell surface display systems (Ishii et al. 2016; Shi et al. 2015) which may be because of the high sugar-tolerant nature of the yeast host strain and different process conditions. Yang et al. (2017) reported that the reusability of TreS from *P. putida* displayed on the cell surface of *P. pastoris* reached three cycles while maintaining a relative conversion rate of 98% compared to the first cycle. However, the reaction durations were prolonged from 16 h in the initial cycle to 25 h in the third cycle in order to attain a trehalose yield comparable to that of the first cycle. More recently, *C. glutamicum* cells expressing TreS from *Streptomyces coelicolor* at the cell surface demonstrated a relative conversion rate of 80% following four cycles of repeated usage while maintaining a rate of 75% after five cycles of reuse (Fang et al. 2023). These observations suggest potential variations in the number of recycling cycles due to the type of TreS enzymes and the expression systems employed.

### Scale-up production of trehalose in a 5-L bioreactor

**Fig. 7:**
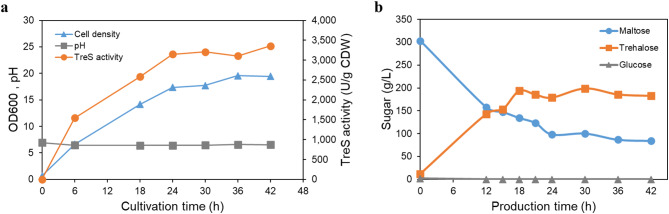
Time course of TreS activity, cell density and pH between fermentation by recombinant *S. cerevisiae* I3A grown in a 5-L fermenter (**a**). Time course of trehalose and maltose concentration by recombinant *S. cerevisiae* I3A in a 5 L fermenter (**b**). Data represent an average of two independent batches

The biotransformation of trehalose from maltose by *S. cerevisiae* strain I3A was scaled up in a 5-L bioreactor containing 3.5 L of fermentation medium. As shown in Fig. 7a, the surface-displayed TreS activity of yeast cells increased over the first 24 h of cultivation and then gradually rose until 42 h. The maximum TreS activity of 3358 U/g CDW and yeast cell concentration of 19.5 OD_600_/mL were reached at 42-h cultivation. The surface-displayed TreS activity of yeast cells was enhanced 1.4-fold when compared with that observed at the flask scale using the fermentation medium. Subsequently, the yeast cells were subjected to trehalose production under optimized conditions in a 5-L bioreactor containing 300 g/L maltose. The scale-up experiments were conducted in two batches. As shown in Fig. 7b, the trehalose concentration continued to increase over time and reached conversion equilibrium after 18 h of incubation. At this time point, the average trehalose concentration was approximately 194 g/L, corresponding to a 64% conversion rate. After 30 h of fermentation, the trehalose concentration rose slightly to 199 g/L, achieving a 66% conversion rate with a residual maltose concentration of 100 g/L. Interestingly, the trehalose productivity reached a maximum of 10.8 g/L/h at 18 h of production time. Extending the reaction time beyond 30 h led to a slight decline in trehalose concentration. This observation suggests a potentially higher rate of the reverse reaction, where trehalose is converted back to maltose.

Our investigation did not detect any glucose by-product during trehalose production by *S. cerevisiae* I3A in a 5-L bioreactor, which is consistent with our previous observations at the flask scale. The HPLC chromatogram of trehalose production by *S. cerevisiae* I3A under optimal conditions at the flask scale revealed no glucose in the reaction solution after 24 h of incubation (Figures S2a, S3). Additionally, the cell density (OD_600_) of *S. cerevisiae* I3A slightly decreased after 12 and 24 h of incubation (Figure S2b). Furthermore, we also investigated trehalose production from maltose using *S. cerevisiae* cells carrying plasmids pYk-KlPIR4-AaTreS, pYk-KlPIR4-PtTreS, and pYk-KlPIR4-PoTreS (Figure S4). The HPLC chromatograms indicated that no glucose was detected for any of the three yeast strains. These results suggest that it is unlikely the yeast consumed glucose. Further studies on whether the displayed AaTreS enzyme produces glucose by-products should be conducted. Notably, among the reported TreS enzymes, only the TreS from *Pseudomonas stutzeri* CJ 38 has been reported to not generate glucose during the catalysis reaction (Lee et al. 2005). The absence of glucose is advantageous as its accumulation can inhibit TreS activity and decrease trehalose yield (Wei et al. 2004; Agarwal and Singh 2021). Furthermore, the presence of only trehalose and maltose in the final solution suggests the potential for a simplified one-stage simulated moving bed chromatography (SMB) for downstream purification, potentially leading to significant cost savings compared to traditional two-stage SMB separation (Song et al. 2016).

The whole-cell biocatalyst constructed using the cell surface display technique eliminates the need for a cell permeabilization step, unlike those employing *E. coli* or *B. subtilis*. (Zheng et al. 2015; Zhu et al. 2020). This CSD method allows for self-immobilization of target enzymes on the yeast cell wall during cell cultivation. (Arnthong et al. 2022). For instance, anchored trehalose synthase from *P. torridus* on the surface of *Y. lipolytica*, achieving a 73% substrate conversion rate and 4.5 g/L/h trehalose productivity (Li et al. 2016) (Table 1). However, their biocatalysis process required a longer reaction time (50 h) compared to our 18-h process. Similarly, another study constructed *P. pastoris* displaying TreS from *P. putida* on the cell surface, yielding up to 64% trehalose and allowing for three recycling cycles, albeit with extended incubation times. While a recent report described displaying a mutant *P. putida* TreS on *B. subtilis* spores with a 74% trehalose conversion rate and the potential for 4 repeated uses with largely retained conversion rate (Liu et al. 2019a). Although *B. subtilis* spore display has shown promise in laboratory studies, its current limitations hinder its translation to commercially viable applications (Wang et al. 2017b). In contrast, our *S. cerevisiae* displaying AaTreS achieved a trehalose production yield of 64% and a productivity of 10.8 g/L/h. The yeast cell surface display technique offers several advantages including safety, ease of process, and industrial feasibility. Notably, *S. cerevisiae* is a non-endotoxin producer, enabling the production of food and pharmaceutical-grade trehalose (Vieira and Delerue-Matos 2020). Nevertheless, further enzyme engineering of AaTreS could potentially improve the conversion rate of maltose-to-trehalose.

**Table 1:** Comparison of trehalose production through different cell and spore surface display techniques

Host strain	Biocatalyst type^1^	Conversion rate (%)^2^	Productivity (g/L/h)	Glucose by-product (g/L)	Fermentation Condition	Reusability (cycle)^3^	Reference
*Y. lipolytica*	CSD	73.0	4.5	~25	50 °C, pH 6	n.d. ^4^	Li et al. (2016)
*P. pastoris*	CSD	64.0	12.0	n.d.	15 °C, pH 8	at least 3	Yang et al. (2017)
*B. subtilis*	SSD	74.1	18.5	n.d.	25 °C, pH 8	at least 4	Liu et al. (2019a)
*C. glutamicum*	CSD	69.5	6.9	~30	35 °C, pH 7	4	Fang et al. (2023)
*S. cerevisiae*	CSD	64.0	10.8	0	40 °C, pH 8	12	This study

^1^ CSD refers to cell surface display; SSD refers to spore surface display

^2^ The conversion rate is based on the percentage of trehalose produced from an initial maltose concentration of 300 g/L

^3^ Recycling round in which the relative conversion rate is greater than 80% of the initial conversion rate

^4^ Not determined

## Conclusion

In summary, the high-sugar tolerant strain of *S. cerevisiae* was identified and used as host cells for the surface-displaying TreS from *A. aeolicum*. The resulting strain demonstrated a high trehalose yield of 64% and a notable productivity of 10.8 g/L/h. The reusability of these cells for up to 12 cycles made the trehalose production process more economical. No glucose was detected during the whole-cell catalysis that facilitated the downstream purification step. These findings highlight the high-sugar tolerant strain of *S. cerevisiae* whole cells as a highly efficient, convenient, practical, and endotoxin-free platform for trehalose production. This system has promising applications for producing high-quality trehalose for both food and pharmaceutical industries.

## Electronic supplementary material

Below is the link to the electronic supplementary material.

Supplementary Material 1

Supplementary Material 2

## Data Availability

All data generated or analyzed during this study are included in this published article (and its supplementary information files).
