# Supplementary material for: Engineering a high-sugar tolerant strain of Saccharomyces cerevisiae for efficient trehalose production using a cell surface display approach
Source: Bioresour Bioprocess. 2024 Oct 18;11(1):101. doi: 10.1186/s40643-024-00816-x (PMC11489382; doi:10.1186/s40643-024-00816-x)
Supplement: Supplementary file 1 — Supplementary Material 1 [file 40643_2024_816_MOESM1_ESM.docx]

**Supporting Information**

**Engineering a high-sugar tolerant strain of *Saccharomyces cerevisiae* for efficient trehalose production using a cell surface display approach**

Kan Tulsook^1^, Piyada Bussadee^1^, Jantima Arnthong^1^, Wuttichai Mhuantong^1^, Panida U-thai^1^, Srisakul Trakarnpaiboon^1^, Verawat Champreda^1^, and Surisa Suwannarangsee^1*^

^1^ National Center for Genetic Engineering and Biotechnology (BIOTEC), National Science and Technology Development Agency (NSTDA), 113 Thailand Science Park, Klong Luang, Pathumthani 12120, Thailand

*** Correspondence:** Dr. Surisa Suwannarangsee Email: surisa.suw@biotec.or.th

**Table S1** Source of TreS enzymes used in this study.

| **TreS enzyme** | **GenBank accession number** | **Source organism for TreS** |
| --- | --- | --- |
| PtTreS | WP_011176870.1 | *Picrophilus torridus* |
| AaTreS | WP_054963950.1 | *Acidiplasma aeolicum* |
| DaTreS | WP_019010376.1 | *Deinococcus aquatilis* |
| DmTreS | WP_027459313.1 | *Deinococcus murrayi* |
| PaTreS | WP_028241539.1 | *Pseudomonas azotifigens* |
| PcTreS | WP_122315296.1 | *Pseudomonas cichorii* |
| PoTreS | WP_084273139.1 | *Picrophilus oshimae* |
| TtTreS | AAQ16097.1 | *Thermus thermophilus* |

**Table S2** List of strains and plasmids used in this study.

| **Strain, plasmid, or primer** | **Description** | **Source/Reference** |
| --- | --- | --- |
| ***Strains*** | | |
| *E. coli* DH5α | F^–^ φ80*lac*ZΔM15 Δ(*lac*ZYA-*arg*F)U169 *rec*A1 *end*A1 *hsd*R17(r_K_^–^, m_K_^+^) *pho*A *sup*E44 λ^–^*thi*-1 *gyr*A96 *rel*A1 | Invitrogen |
| *S. cerevisiae* TBRC 3611 | Wild type strain | TBRC^1^ |
| *S. cerevisiae* TBRC3611-Ade2∆ | *Ade2* auxotrophic strain of TBRC 3611 | This study |
| *S. cerevisiae* I3A | *S. cerevisiae* TBRC3611-Ade2∆ containing GPD1_p_-SS_SUC2_-KlPIR4-AaTreS-CYC1_T_ integration cassettes | This study |
| ***Plasmids*** | | |
| pYES3-Kan | pYES3/CT derivative with G418 resistance gene (KanMX) as a selectable marker | Laboratory collection |
| pYk-KlPIR4-PtTreS | pYES3-Kan GPD1_p_-SS_SUC2_-KlPIR4-PtTreS-CYC1_T_ | This study |
| pYk-KlPIR4-AaTreS | pYES3-Kan GPD1_p_-SS_SUC2_-KlPIR4-AaTreS-CYC1_T_ | This study |
| pYk-KlPIR4-DaTreS | pYES3-Kan GPD1_p_-SS_SUC2_-KlPIR4-DaTreS-CYC1_T_ | This study |
| pYk-KlPIR4-DmTreS | pYES3-Kan GPD1_p_-SS_SUC2_-KlPIR4-DmTreS-CYC1_T_ | This study |
| pYk-KlPIR4-PaTreS | pYES3-Kan GPD1_p_-SS_SUC2_-KlPIR4-PaTreS-CYC1_T_ | This study |
| pYk-KlPIR4-PcTreS | pYES3-Kan GPD1_p_-SS_SUC2_-KlPIR4-PcTreS-CYC1_T_ | This study |
| pYk-KlPIR4-PoTreS | pYES3-Kan GPD1_p_-SS_SUC2_-KlPIR4-PoTreS-CYC1_T_ | This study |
| pYk-KlPIR4-TtTreS | pYES3-Kan GPD1_p_-SS_SUC2_-KlPIR4-TtTreS-CYC1_T_ | This study |
| pYIR3 | A multi-copy integrative plasmid with an *ADE2* marker | Laboratory collection |
| pYIR3-AaTreS | pYIR3 GPD1_p_-SS_SUC2_-KlPIR4-AaTreS-CYC1_T_ | This study |

^1^ TBRC refers to Thailand Bioresource Research Center (TBRC, www.tbrcnetwork.org).

**Table S3** Primers used in this study.

| Primer | Sequences (5’->3’) |
| --- | --- |
| Col_pYES3-kan_R | AGCGTCCCAAAACCTTCTCAAGCA |
| Col_pYES3-kan_F | CCACTACGTGAACCATCACCCTAATCAA |
| Col_KanMX_F | GACTTGATCTAGAGACATGGAGGCCCAGAATACCC |
| Col_KanMX_R | AGCTACGCGGCCGCCAGTATAGCGACCAGCATTC |
| Col_pYIR_F | ATGCAATGTATACTAAACTCACAAA |
| Col_pYIR_R | GGCTGCAAAACCAGCCAAA |

**
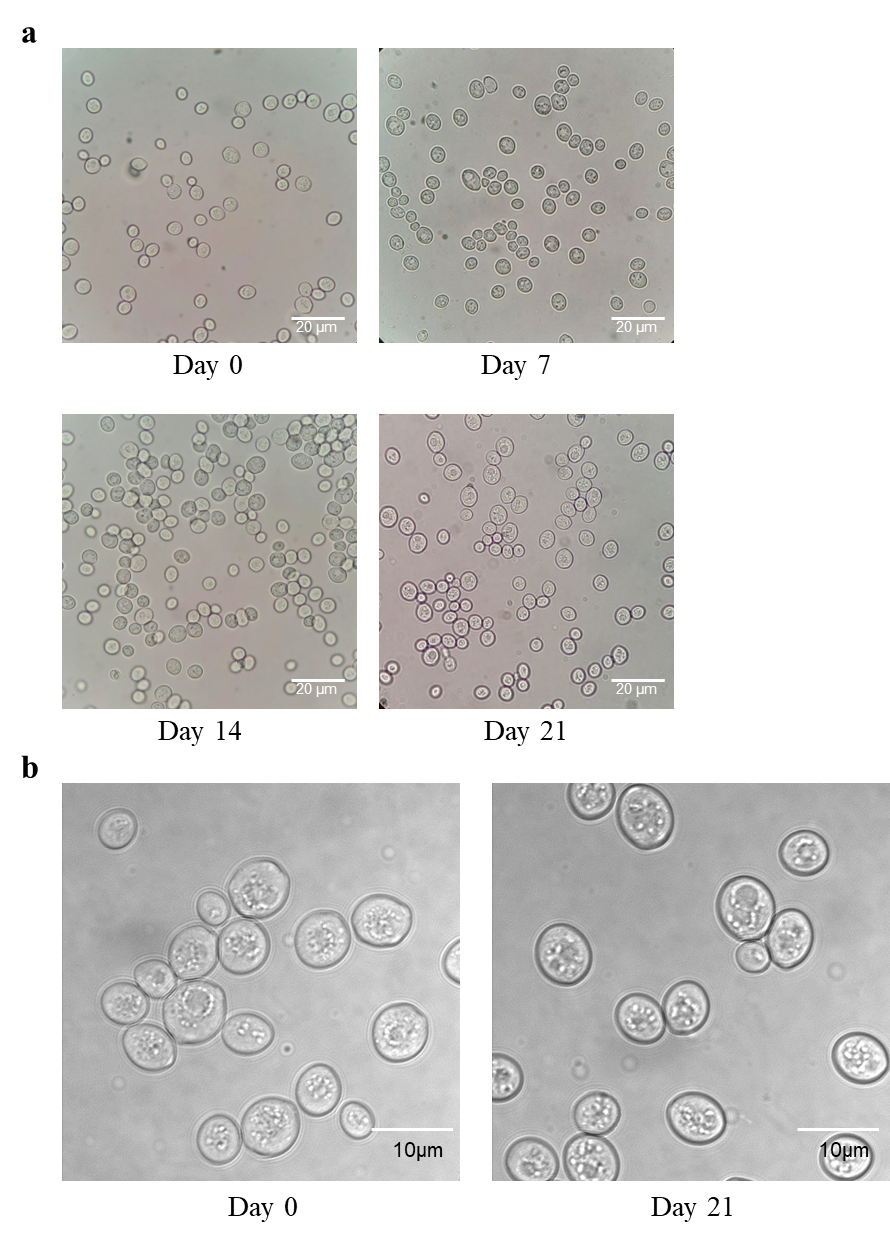
**

**Figure S1.** Light micrographs of the *S. cerevisiae* I3A cells after storage in 50 mM sodium phosphate buffer solution at pH 8.0 at 4 °C for 0, 7, 14, and 21 days. Cells were imaged using a ×40 objective lens (a) and ×100 oil immersion objective lens (b).


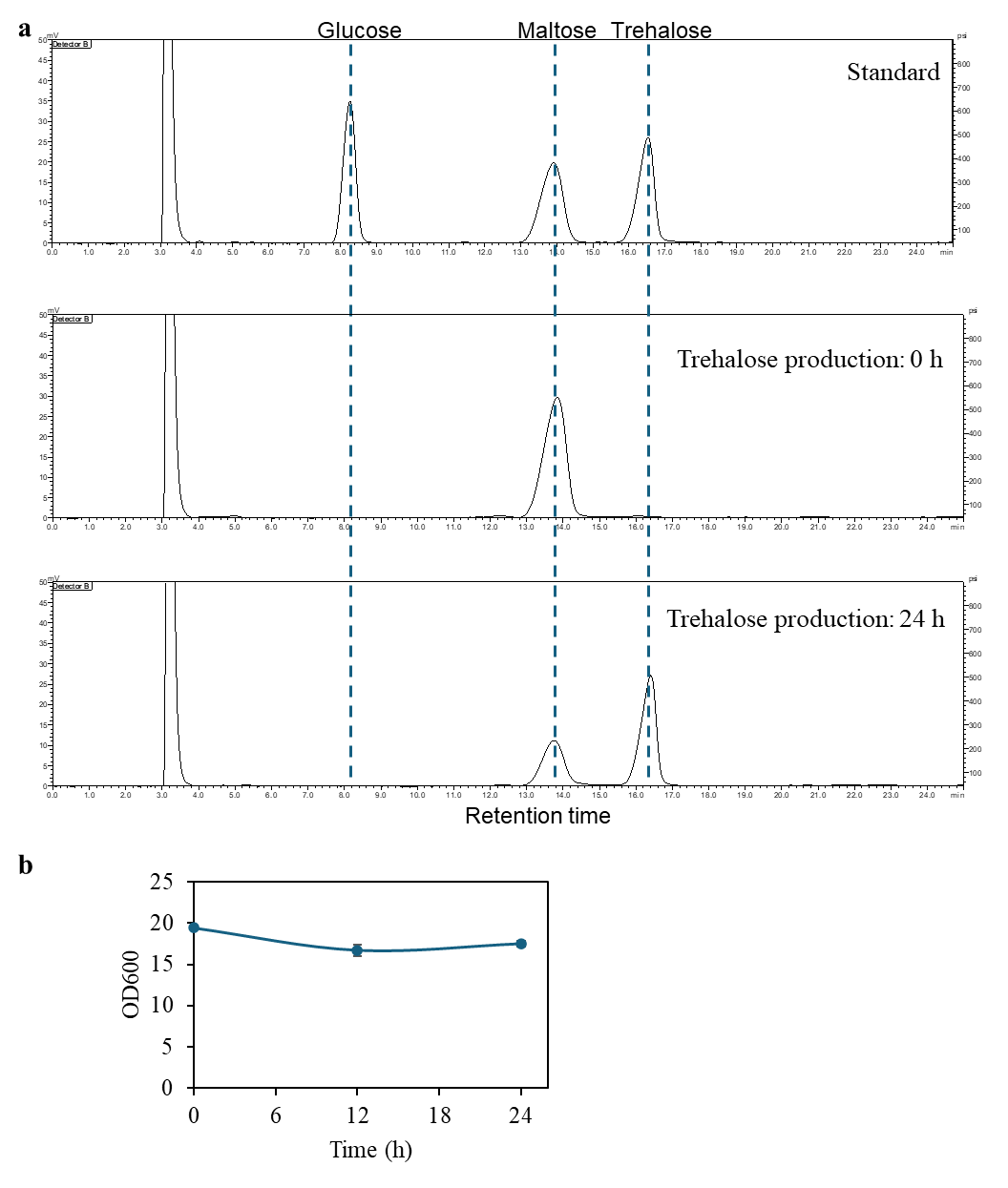


**Figure S2.** (a) HPLC chromatograms of sugar standards (concentration of each sugar is 10 g/L) and samples of trehalose production by *S. cerevisiae* I3A cells at optimum conditions after incubation for 0 and 24 h. Samples were diluted 20 folds with distilled water. (b) Cell density (OD_600_) of *S. cerevisiae* I3A cells during trehalose production at 0, 12, and 24 h. The trehalose production was carried out at optimal conditions (300 g/L of maltose, 20 OD_600_ of yeast cells, 40 °C, 200 rpm). Results represent the means ± standard deviation (SD) of three independent experiments.


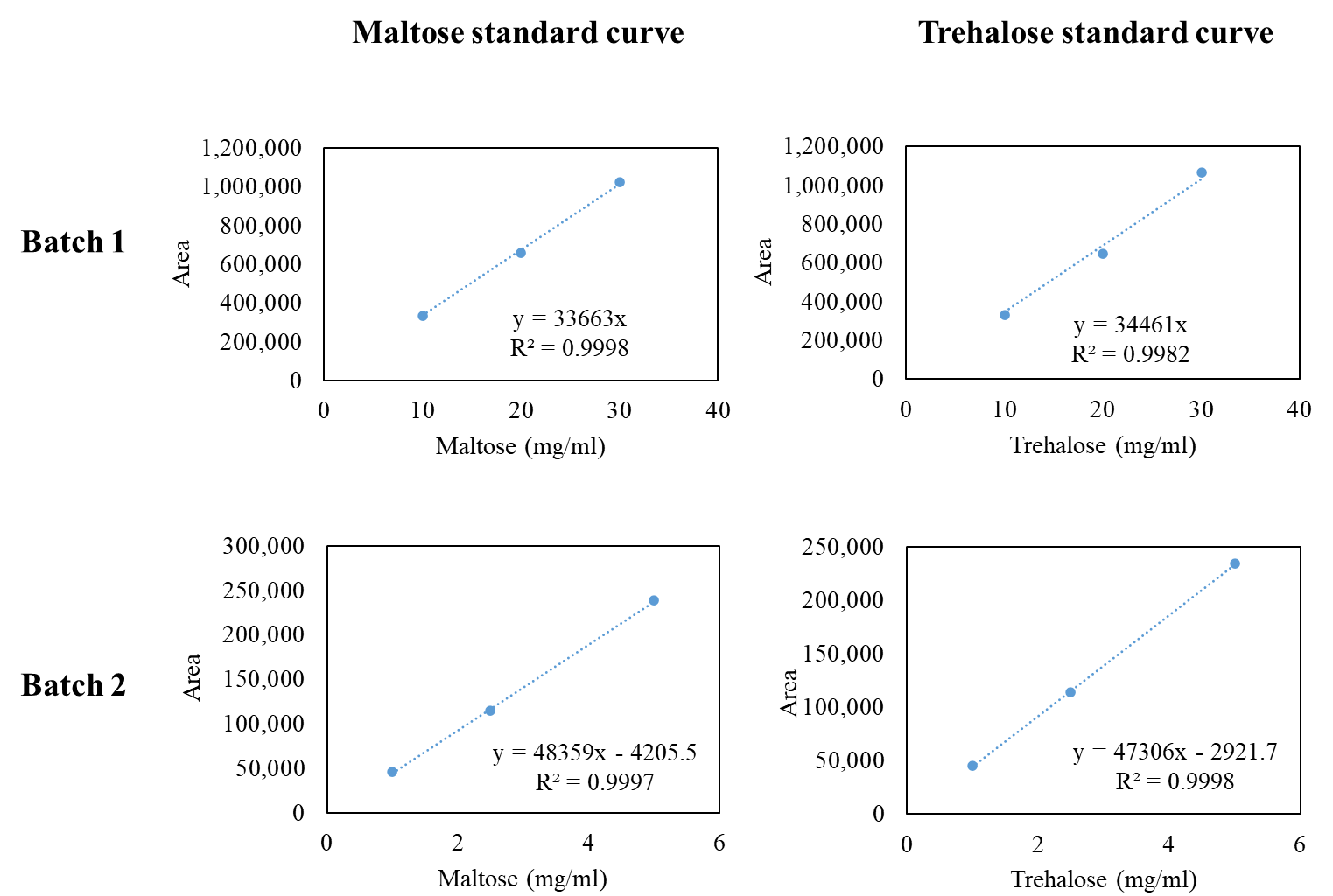


**Figure S3.** HPLC standard curve of maltose and trehalose used in trehalose production in 5-L bioreactor.

**
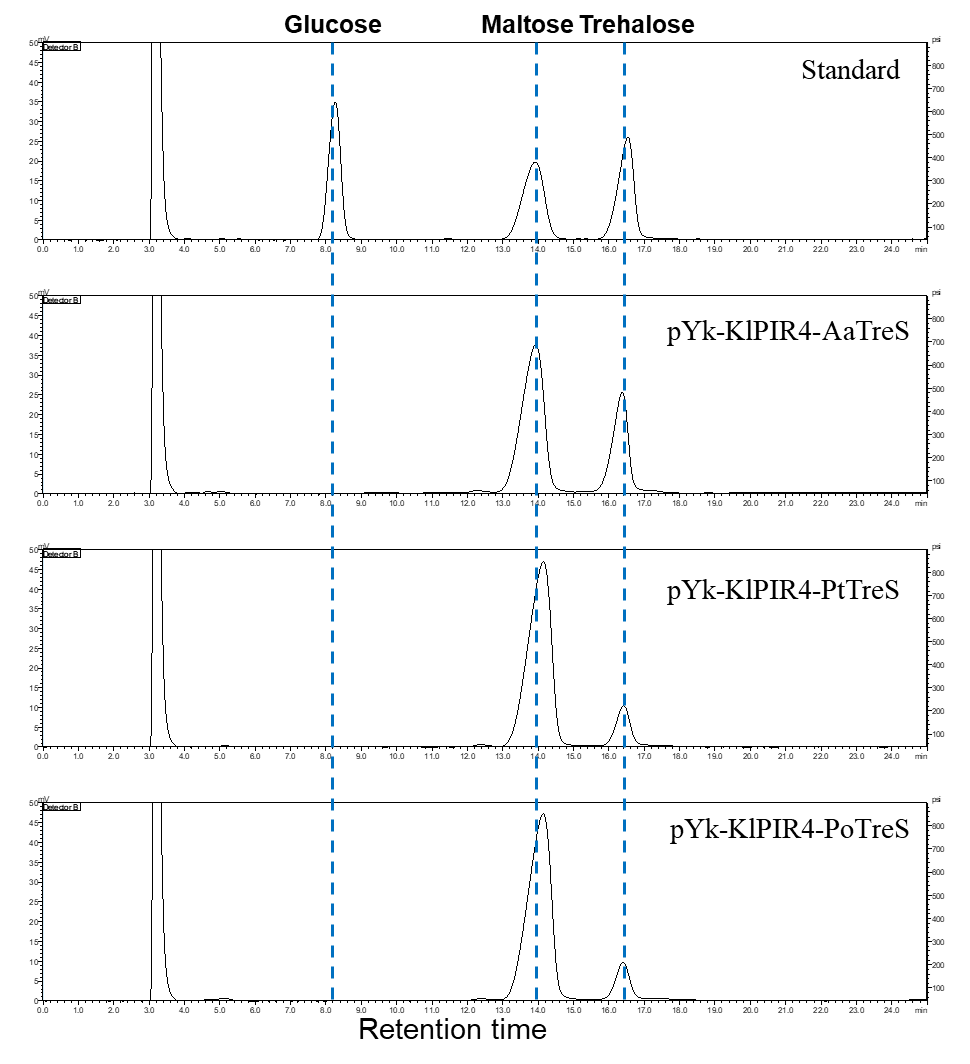
**

**Figure S4.** HPLC chromatograms of sugar standards (concentration of each sugar is 10 g/L) and samples of trehalose production from 300 g/L maltose by *S. cerevisiae* cells carrying pYk-KlPIR4-AaTreS, pYk-KlPIR4-PtTreS, and pYk-KlPIR4-PoTreS after incubation at 40 °C, 200 rpm for 24 h. Samples were diluted 10 folds with distilled water.
