# Supplementary figures and images for: Engineering a high-sugar tolerant strain of Saccharomyces cerevisiae for efficient trehalose production using a cell surface display approach
Source: Bioresour Bioprocess. 2024 Oct 18;11(1):101. doi: 10.1186/s40643-024-00816-x (PMC11489382; doi:10.1186/s40643-024-00816-x)

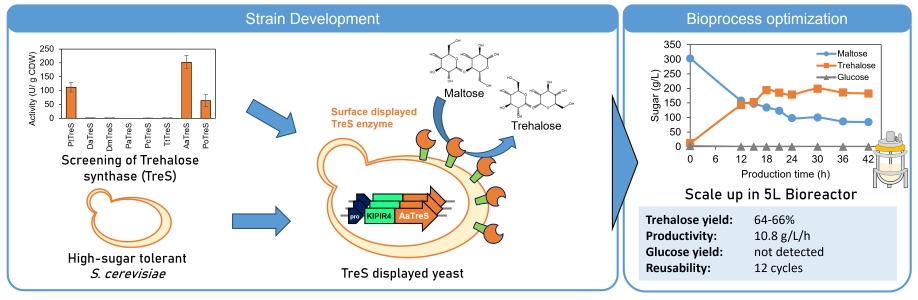

Supplement: Supplementary file 2 — Supplementary Material 2 [file 40643_2024_816_MOESM2_ESM.jpg]
